# Eighth Val-de-Grâce Emerging Infectious Diseases Seminar, Paris, France, March 29, 2019

**DOI:** 10.3201/eid2603.191386

**Published:** 2020-03

**Authors:** Paul Le Turnier, Jocelyn Raude, Catherine Leport, Corinne Jadand, Bruno Hoen, Didier Che, Sylvie Sargueil, Patrick Zylberman, Jean-François Guégan

**Affiliations:** Centre Hospitalier Universitaire de Nantes, Nantes, France (P. Le Turnier);; Unité des Virus Emergents, Marseille, France (J. Raude);; École des Hautes Etudes en Santé Publique Rennes, Université Sorbonne Paris Cité, Paris, France (J. Raude, P. Zylberman);; Unité des Virus Emergents, Aix-Marseille Université, IRD, Inserm, IHU Méditerranée Infection, Marseille, France (J. Raude, P. Zylberman);; Université Paris Diderot, Inserm, Paris (C. Leport);; Assistance Publique-Hôpitaux de Paris, Paris, France (C. Leport, C. Jadand);; Centre Hospitalier Universitaire de Nancy, Nancy, France (B. Hoen);; Santé Publique France, Saint-Maurice, France (D. Che); Independent health journalist, Paris (S. Sargueil);; École des Hautes Etudes en Santé Publique, Rennes, France (P. Zylberman);; MIVEGEC, IRD, CNRS, Université de Montpellier, Montpellier, France (J.-F. Guégan);; ASTRE, INRA, Cirad, Université de Montpellier, Montpellier, France (J.-F. Guégan);; oneHEALTH Global Research Programme, FutureEarth Programme, Paris (J.-F. Guégan)

**Keywords:** Emerging infectious disease, communication, management, disease risk, real-time adaptation, effective epidemic response, Val-de-Grâce Emerging Infectious Diseases Seminar, Paris, France

The eighth Val-de-Grâce School Seminar on Emerging Infectious Diseases was held in Paris, France, on March 29, 2019. The goal of this annual seminar is to bring together scientists, practitioners, experts, and decision makers interested in human, animal, and plant health (the One Health approach) but also to extend it to social sciences, environmental sciences, prospective analyses, mathematical modeling, biosafety, and defense. Bedford and colleagues recently emphasized the need for an interdisciplinary approach during emerging infectious disease management (*1*). Health communication has been recognized as a key tool to use against new epidemics and pandemics (*2*). Health communication can take many forms, from public and digital communications to traditional and interpersonal communications (*3*). In the field of emerging infectious diseases, communication generally refers to interventions by which a set of key facts and skills is identified, selected, and shared with individuals, groups, or organizations to promote an effective collective response (*4*). Implementation of communication methods and strategies by experts during emerging infectious disease situations is challenging, notably because of the diversity of the audience (e.g., decision makers, health professionals, general public, and specific populations). This seminar addressed the communication challenges encountered during emerging infectious disease events, focusing on communication from experts in science and medicine.

The seminar opened with a lecture by Pierre Le Coz, entitled “Communicating within Uncertainty.” Using examples dating back to ancient times, he reminded the audience how difficult sending rational messages may be when it comes to humans being endowed with imagination and having concerns and feelings. Transmitting rational messages may be helped by appropriate timing, trustworthiness, and realism.

Of the 2 panel discussions, the first was “Communication from Field Actors to Decision Makers.” The first speakers were Bruno Hoen and Patrick Saint-Martin, who discussed “Feedback on the Zika Experience in the French West Indies in 2016.” They started by briefly summarizing the Zika outbreak in the French West Indies and describing how communication was prepared even before the first cases were identified. A community-based strategy was set up at several levels to increase the chances of reaching the most vulnerable persons, especially pregnant women. Despite this anticipation, the communication strategy had to be adjusted according to the data and knowledge available at the time of the outbreak and the perception of prevention messages. Moreover, the speakers pointed out that as the incidence of the disease decreased, the French West Indies population felt less involved and receptivity to health messages waned, justifying real-time adjustment of the communication strategy by regional and national health authorities.

Simon Cauchemez then presented “Mathematical Models: Making the Results Understandable.” He discussed the objectives of those mathematical models, namely, contextualizing an epidemic phenomenon and explaining the determinants of an existing situation at any given time. This approach can help predict the evolution of an outbreak and anticipate operational control and prevention strategies. He emphasized that these real-time models are limited by the availability of field data during the epidemic. Therefore, uncertainty of these results should be kept in mind, especially by decision makers, and the links between surveillance, care, research, and health promoters should be strengthened.

Patrick Brasseur presented “Crisis Communication: Principles, Imperatives, and Needs of Decision Makers.” He described a communication coordinator’s role when dealing with a decision maker and listed what a decision maker needs, or does not need, to communicate. He emphasized the 4 principles established in the World Health Organization guidelines for emergency risk communication policy and practice: trust, transparency, consistency, and acceptance of uncertainty (https://apps.who.int/iris/bitstream/handle/10665/259807/9789241550208-eng.pdf?sequence=2) He pointed out the limits of the measures whose sole objective is reassurance and the risk for a backfire effect. Last, he emphasized that communication is often tailored to a given geographic context and cannot be transposed without appropriate adjustments.

The second panel discussion—“What to say? To whom? When and How?”—addressed those questions. When discussing “Institutional Communication,” David Heard stated that providing information without an appropriate communication strategy is generally ineffective and sometimes counterproductive, as demonstrated in vaccine promotion studies (*5*). The issue of communication was addressed, including the crisis communication plan. Also addressed were the choice of the channels best suited to communicating and the notion of listening to the response from the targeted audience. The relevance of and modalities for evaluating communication after a crisis were also thoroughly discussed with participants.

Bernard Seytre presented “What is Health Literacy? Building on the Knowledge of the Individuals to Change Behavior.” Health literacy consists of promoting changes in social behavior toward greater autonomy and better health. To do this, information receivers must be defined beforehand, after which an accurate assessment of the current situation and previously failed communication strategies is needed to develop better-adapted methods. As an example, Seytre used the instructions to reduce wildlife hunting to combat the spread of the Ebola virus in Africa, which he considered irrelevant in view of the modes of transmission during epidemic periods, impracticable given the predominant role of bush meat in some African regions, and potentially counterproductive for communication toward at-risk communities.

Also in the second panel, Arnaud Veïsse and Joseph Rustico presented “Information for Target Populations (Example of Refugees).” They described the difficulties of targeting particularly hard-to-reach vulnerable persons, such as asylum seekers. To establish relevant and efficient communication with these vulnerable groups, inclusion of specific frailty parameters (e.g., language difficulties, traumatic experiences, insecure legal status) is necessary. He discussed the role of the health mediator as a potentially effective solution to foster reciprocal understanding between vulnerable persons, their relatives, and the institutions (e.g., the health system).

Undoubtedly, communication from experts represents a key area for emerging infectious disease crisis management. Over time, communication should be adapted to the evolving epidemiologic situation and to the profile of targeted populations. Its many limitations must be known and anticipated by decision makers. Other sources of communication (e.g., journalists, social networks) could also play a role during an emerging infectious disease crisis and should be the subject of further analysis.
